# The effect of Lasik surgery on myopic anisometropes’ sensory eye dominance

**DOI:** 10.1038/s41598-017-03553-8

**Published:** 2017-06-15

**Authors:** Lixia Feng, Huimin Lin, Yao Chen, Jiafeng Wang, Yonghua Wang, Rongfeng Liao, Jiawei Zhou, Robert F. Hess

**Affiliations:** 1 Department of Ophthalmology, First Affiliated Hospital, Anhui Medical University, Hefei, Anhui P.R. China; 2 School of Ophthalmology and Optometry and Eye hospital, Wenzhou Medical University, Wenzhou, Zhejiang P.R. China; 3 State Key Laboratory of Ophthalmology, Optometry and Vision Science, Wenzhou Medical University, Wenzhou, Zhejiang P.R. China; 4 McGill Vision Research, Dept. Ophthalmology, McGill University, Montreal, PQ Canada

## Abstract

Lasik is a common surgery for treating anisometropia. In this study, we asked a specific question: *what*’*s the effect of Lasik surgery on anisometropes*’ *sensory eye dominance* *?* Fifteen myopic anisometropes (mean age: 23 ± 6.9 years old; 6 females) participated in our experiment. We quantitatively measured participants’ sensory eye dominance before and after the Lasik surgery using a binocular phase combination paradigm. We found no significant change of sensory eye dominance within 16 weeks (measured between 8 to 96 days, for one or two repetitions) after the surgery (t(14) = −1.44, *p* = 0.17). A further following on eight patients showed that patients’ two eyes were much more balanced at 16 weeks or more (measured one or two times between 112 to 408 days) after the surgery (t(7) = −3.79, *p* = 0.007). Our results suggest that the benefit of Lasik surgery on anisometropes’ sensory eye dominance is not immediate, a long-term ‘adaptation’ period (16 weeks or more) is necessary to enable the surgery to be truly effective.

## Introduction

Myopia is a common prevalent disorder, such a problem is particularly pronounced^1^ and affecting up to 90% of teenagers and young adults in China^2^. In some cases, patients’ two eyes have different degree of myopia, i.e, myopic anisometropia^3, 4^. Laser *in-situ* keratomileusis (LASIK) has become a popular method to treat anisometropia. Several postoperative evaluation studies have been conducted, which showed that LASIK surgery is safe and effective in reducing patients’ spherical equivalent manifest refraction and increasing their visual acuity^5^, but may increase the 3rd and higher order aberrations^6^ or there may be a risk of postoperative diplopia^7^.

On the other hand, anisometropia has been suggested to be an important cause of abnormal binocularity^8, 9^. Recently, Zhou *et al*.^10^ also showed that patients who are corrected for myopic anisometropia have abnormal sensory eye dominance, but that this improves during 16 or more weeks of refractive adaptation. Their results suggest that refractive adaptation may play a critical role in partially restoring the sensory eye balance in corrected anisometropes. In spite of many previous studies in anisometropia and binocularity, to our knowledge, there is no direct information on how sensory eye balance is affected after LASIK surgery. Therefore, we conducted a cohort study to quantitatively evaluate the postoperative effect of LASIK surgery on myopic anisometropes’ sensory eye dominance.

## Results

**Figure 1:**
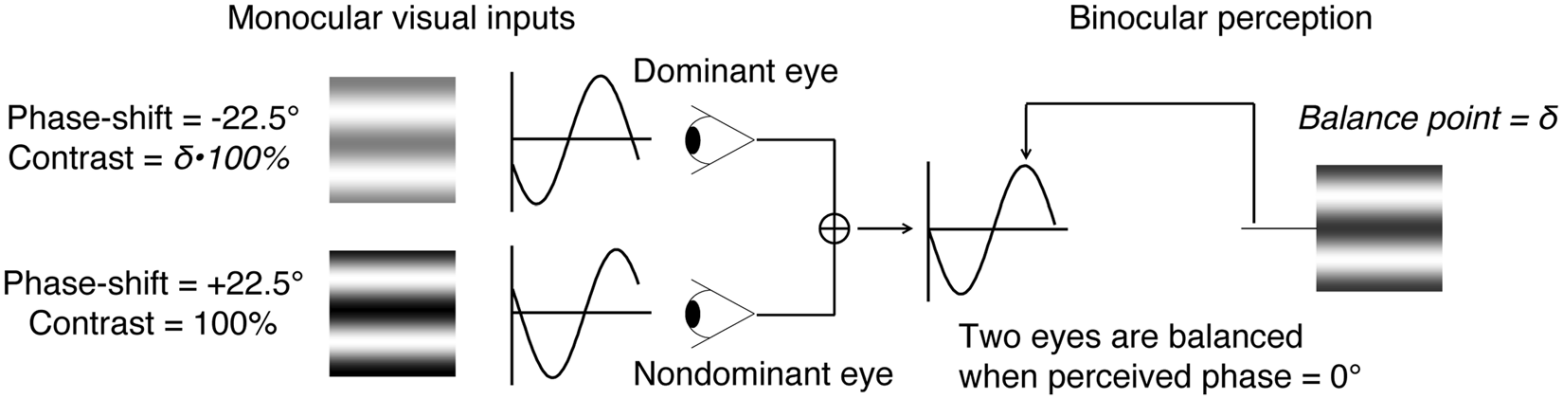
Illustration of the binocular phase combination paradigm. Two horizontal sine-wave gratings with equal and opposite phase-shifts of 22.5° (relative to the center of the screen) were dichoptically presented to the two eyes through the polarized glasses. The perceived phase of the cyclopean grating depends on the contribution of the two eyes in binocular phase combination. Sensory eye dominance is quantified by the interocular contrast difference that is needed to achieve a 0-degree of perceived phase, i.e., the balance point, where the two eyes are balanced in binocular phase combination.

**Figure 2:**
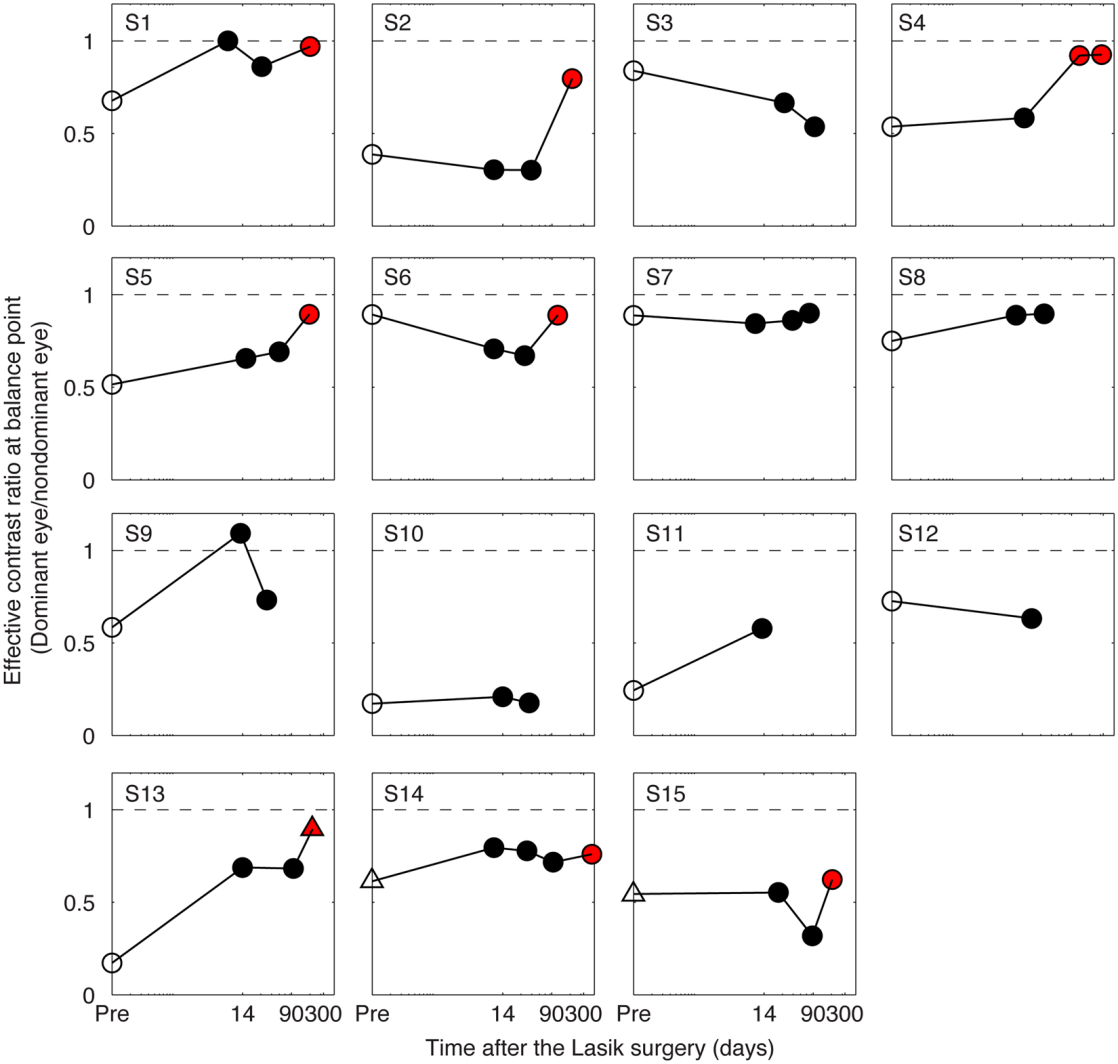
Patients’ sensory eye dominance before and after the surgery. Patients’ sensory eye dominance were quantified by individuals’ effective contrast ratio at balance point (i.e., the interocular contrast ratio that is needed when the two eyes are balanced in binocular phase combination) before and after surgery. The pre-surgery measurement is marked as open symbols; the post-surgery measurement that was measured within 16 weeks after the surgery is marked as black filled symbols and the post-surgery measurement that was measured at or longer than 16 weeks after the surgery is marked as red filled symbols. Except subjects S13, S14 and S15, who had a switch of eye dominance in one of the test sessions (marked as triangle symbols), most observers had consistent nondominant eye during this study.

In the experiment, we used a binocular phase combination paradigm to quantitatively access the sensory eye dominance of our participates. In particular, observers’ sensory eye dominance was quantified by the interocular contrast ratio that was needed when the two eyes were balanced in binocular phase combination (Fig. 1). Figure 2 shows individuals’ effective contrast ratio corresponding to their balance points before and after surgery. As is shown in Fig. 2, all the patients’ pre-surgery balance points (marked as open symbols) were lower than the ideal normal level (i.e., 1.0; marked as dashed line in each panel). This result is consistent with a recent report^10^, which suggests that anisometropes exhibit interocular sensory imbalances. Within 16 weeks after the surgery, the balance points (marked as black filled symbols) didn’t change much for most of the patients. A longer-term follow up in eight patients showed that patients’ balance points (marked as red filled symbols) were much closed to the ideal normal level, indicating improved interocular balance.

**Figure 3:**
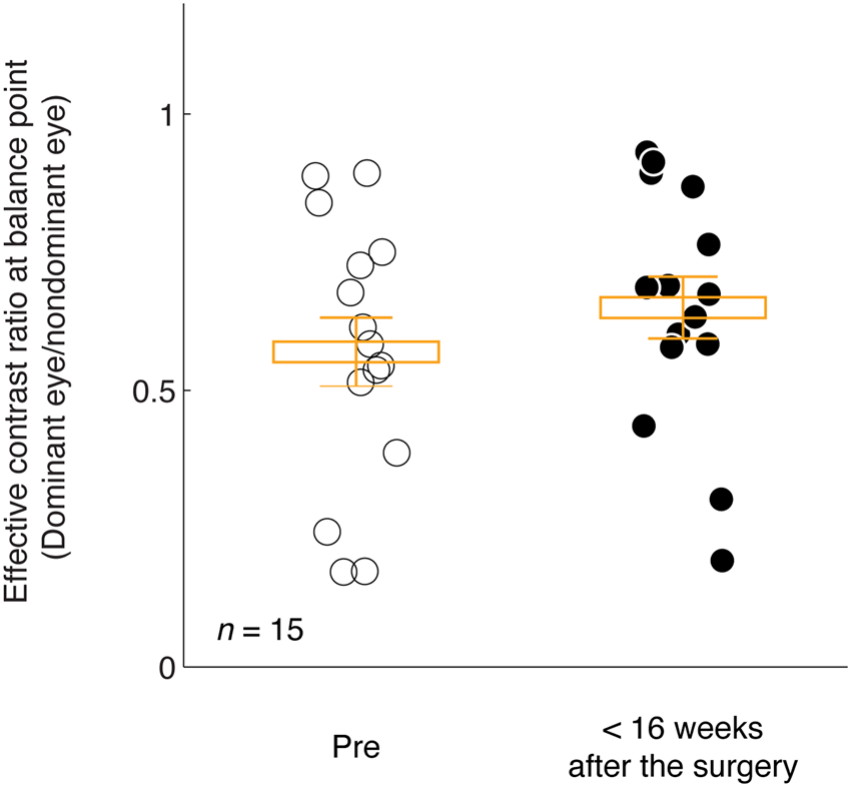
Sensory eye dominance before and within 16 weeks after the surgery. Each point represents the results of one subject. The average balance point and it’s standard error were marked using box and error bars for each time session.

To better show the change of balance points within 16 weeks after the surgery, we plotted individuals’ balance points before and after surgery in Fig. 3. It is obvious that the balance points within 16 weeks after the surgery were consistent with that of pre-surgery. A 2-tailed paired-samples t-test also show that there was no significant change of balance points within 16 weeks after the surgery: t(14) = −1.44, *p* = 0.17.

**Figure 4:**
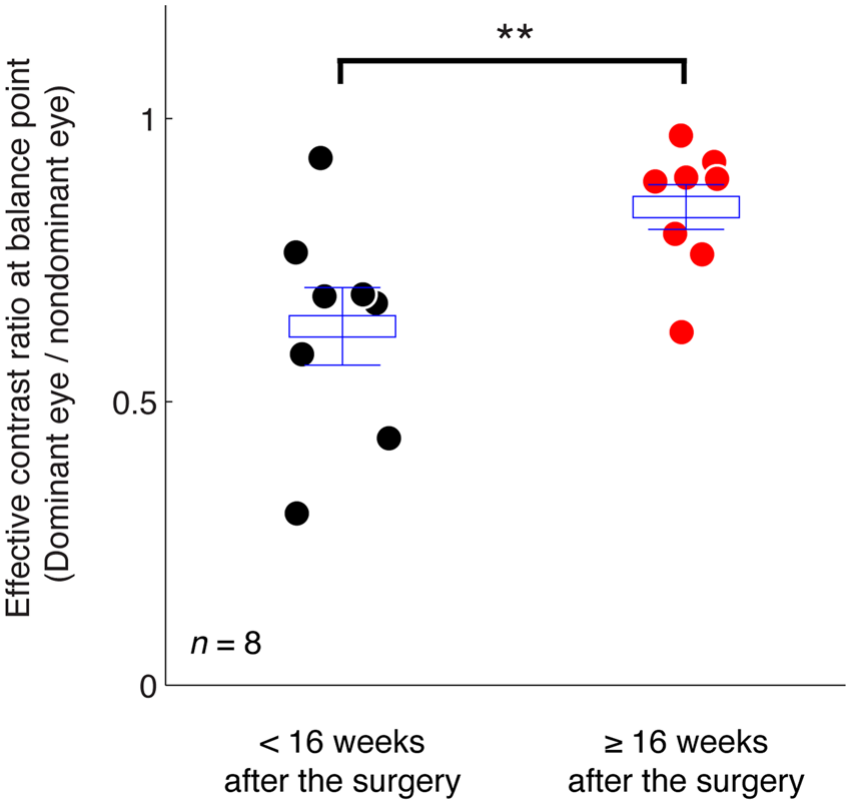
The long-term effect of surgery on patients’ sensory eye dominance. Each point represents the results of one subject. The average balance point and it’s standard error were marked using box and error bars for each time session. Eight patients participated in this study. **p < 0.01, 2-tailed paired-samples t-test.

To better illustrate the long-term effect of surgery on patients’ sensory eye dominance, we plotted patients’ balance points that were measured within 16 weeks after the surgery compared with those measured at 16 weeks and longer after the surgery in Fig. 4. Eight patients participated in this additional long-term following study. Most of the patients had an improved balance point at or longer than 16 weeks after the surgery. A 2-tailed paired-samples t-test also show that there was a significant change of balance points at these two time sessions: t(7) = −3.79, *p* = 0.007.

## Discussion

We show that LASIK surgery doesn’t produce an immediate effect on myopic anisometropes’ sensory eye dominance; the postoperative effect needs 16 weeks or more to be effective. It should be noted that while the first part of conclusions was derived from 15 myopic anisometropes’ results (Fig. 3), the second part of conclusions was derived from only 8 myopic anisometropes’ results (Fig. 4), as other patients didn’t participated in this long-term following due to personal reasons. We are therefore limited in what we can conclude about postoperative changes in all anisometropes. However, what we can conclude is that LASIK surgery doesn’t produce an immediate effect on myopic anisometropes’ sensory eye dominance. Nevertheless, our results are consistent with the recent report by Zhou *et al*.^10^, suggesting that a period of refractive adaptation (>16 weeks) is critical after the correction of anisometropia for improved interocular balance.

We noticed that, even after 16 or more weeks, there were still some patients who had abnormal sensory eye dominance (balance point less than 0.9). It is possible that in these cases that a longer period of refractive adaptation was required or alternatively the remaining eye imbalance may remain permanently after Lasik surgery. On the basis of our previous results on optically corrected anisometropes^10^ and what is known of refractive adaptation^11^, the latter conclusion is the more likely. Such a residual eye imbalance is more likely to be due to a neuronal change in ocular dominance than have a purely optical explanation as any interocular differences in higher order aberrations not corrected by or induced by Lasik surgery^12^ should involve mid to high spatial frequencies^13^ (>5 c/d) and not the very low spatial frequency (1 c/d) used for these measurements of eye balance.

In future studies, it will be interesting to see whether the residual imbalances in ocular dominance that remain after 16 weeks of refractive adaptation can recover after much longer duration of refractive adaptation, or whether they could be able to be rectified by a dichoptic training protocol that has proven successful in rebalancing the eyes of amblyopic adults. It involves playing dichoptic videogames^14–16^ or watching dichoptic movies^17^ where the contrast balance between the eyes is adjusted over time. Typically a 2–6 week period of 1 hr a day is sufficient to balance the eyes and provide better binocular vision and improved stereopsis.

## Methods

### Participants

Fifteen myopic anisometropes (mean age: 23 ± 6.9 years old; 6 females) participated in our experiment. They were recruited from the department of Ophthalmology of the First Affiliated Hospital of Anhui Medical University (Anhui, China). Anisometropia is defined as a 1.50D or larger interocular spherical difference. All participants have normal or corrected to normal visual acuity (LogMAR < 0.10) in the two eyes, no strabismus and no history of eye pathology before the study. Clinical details of patients before surgery are provided in Table 1. Individuals’ sensory eye dominance was assessed before and after the surgery at different time points. Observers wore their prescribed optical correction, if needed, for the data collection.

**Table 1:** Clinical details of the participants.

Subject#	Age (years old)	Gender	Right eye			Left eye		
			Refractive errors	Visual acuity (LogMAR)		Refractive errors	Visual acuity (LogMAR)	
				Uncorrected	Corrected		Uncorrected	Corrected
S1	18	Male	−4.25	0.70	0.00	−1.75	0.52	0.00
S2	19	Male	−4.50/−1.25@10	1.10	0.00	−9.50/−3.25@155	1.70	0.00
S3	20	Male	−3.75	0.92	0.00	−5.75	1.10	0.00
S4	18	Male	−5.75/−1.25@15	1.22	0.00	−3.75/−2.50@170	1.00	0.10
S5	18	Female	−5.25/−0.25@15	0.92	0.00	−3.00/−0.50@10	0.92	0.00
S6	27	Male	−3.25/−1.00@180	0.92	0.00	−5.50/−0.50@10	1.30	0.00
S7	19	Female	−1.00	0.70	0.00	−7.00/−0.50@100	1.70	0.00
S8	20	Male	−4.00/−3.25@10	1.70	0.00	−7.00/−2.75@5	1.70	0.00
S9	32	Female	−6.00/−1.75@170	1.00	0.10	−11.00/−1.50@5	1.22	0.10
S10	43	Female	Plano	0.10	0.10	−3.50/−1.00@160	1.00	0.10
S11	25	Male	−1.25/−3.00@180	0.60	0.10	−3.50/−4.00@170	0.60	0.10
S12	23	Male	−2.75/−1.00@105	0.92	0.00	Plano	0.00	0.00
S13	25	Female	−9.00/−0.50@90	1.52	0.00	Plano	0.10	0.10
S14	20	Female	−6.75/−1.75@150	1.40	0.00	−4.25/−1.25@10	1.30	0.00
S15	18	Male	−5.50	1.00	0.00	−9.00/−1.75@170	1.00	0.00

All subjects were naive as to the purpose of the experiment. A written informed consent was obtained from each of them after explanation of the nature and possible consequences of the study. This study complied with the Declaration of Helsinki and was approved by the Institutional Review Boards of Anhui Medical University, Wenzhou Medical University and McGill University.

### Apparatus

All measurements were conducted on a PC computer running Matlab (MathWorks, Inc., Natick, MA) with PsychToolBox 3.0.9 extensions^18, 19^. The stimuli were presented on a gamma-corrected LG D2342PY 3D LED screen (LG Life Science, Korea) with a 1920 × 1080 resolution and a 60 Hz refresh rate. Subjects viewed the display dichoptically with polar glasses in a dimly lit room at a viewing distance of 136 cm. The background luminance was 46.2 cd/m^2^ on the screen and 18.8 cd/m^2^ through the polar glasses. A chin-forehead rest was used to minimize head movements during the experiment.

### Lasik Surgery

All the patients underwent a series examination before femto-LASIK surgery. The examination included uncorrected distance visual acuity, slit-lamp microscopy, corneal topography, manifest and cycloplegic refraction, corrected distance visual acuity and fundus examination. All the surgeries were performed by the fifth author of this paper (Dr. Liao). During this research, WaveLight FS200 femtosecond laser was used to create superior hinged flaps, with 110 µm thickness and 8.5 mm diameter for the myopic eyes. After the flap was lifted, ablations were performed using Alcon WaveLight EX200 excimer laser (Fort Worth, TX, USA) with a 0.5-mm transition zone and 6.0-mm optical zone.

### Design

We quantitatively accessed all the 15 anisometropes’ sensory eye dominance before the surgery and within 16 weeks after the surgery. The time for post-surgery measure ranged from 8 to 96 days for one or two measurement repetitions at the patients’ convenience. Eight patients participated in an additional, long-term follow up investigation at 16 weeks or longer after the surgery, ranged from 112 to 408 days (due to personal reasons, other patients didn’t participated in this long-term following).

A binocular phase combination paradigm^20, 21^ was used for measuring sensory eye dominance. The design was same as the one we used in previous studies^22–24^, in which observers were asked to dichoptically view two horizontal sine-wave gratings (spatial frequency: 1 cycle/°; size: 2° × 2°) having equal and opposite phase-shifts of 22.5° (relative to the center of the screen) through polarized glasses; the contrast of the nondominant eye was fixed at 100% and the contrast of the dominant eye was varied by an interocular contrast ratio *δ* = [0, 0.1, 0.2, 0.4, 0.8, 1.0] in different trials; the perceived phase of the grating in the cyclopean percept was measured as a function of the interocular contrast ratio. By this method, we were able to find a specific interocular contrast ratio where the perceived phase of the cyclopean grating was 0, indicating equal contribution of the two eyes to the binocular percept. This specific interocular contrast ratio is termed the “balance point” for binocular phase combination since the two eyes under these stimulus conditions are balanced in binocular viewing (Fig. 1). Similar to previous studies^20–26^, two configurations were used for each interocular contrast ratio in the measurement so that any potential positional bias will be cancelled out: in one configuration, the phase-shift was +22.5° in the nondominant eye and −22.5° in the dominant eye and in the other, the reverse. The perceived phase of the cyclopean grating at each interocular contrast ratio (δ) was quantified by half of the difference between the measured perceived phases in these two configurations. Different conditions (configurations and interocular contrast ratios) were randomized in different trials. The perceived phase and its standard error were calculated based on eight measurement repetitions.

Before the start of data collection, proper demonstrations of the task were provided by practice trials to ensure observers understood the task. The nondominant eye was selected based on individuals’ performance in the practice session: the eye that was less dominant in the binocular phase combination task when the two eye had same strength of input (i.e., 100% of contrast) was chosen as the nondominant eye and would have a constant contrast input of 100% during the test. Except subjects S13, S14 and S15, who had a switch of eye dominance in one of the test sessions (marked as triangle symbols in Fig. 2), most observers had consistent nondominant eye during this study.

### Procedure

We used the same phase adjustment procedure as used by Huang *et al*.^21^ for measuring the perceived phase of the binocularly combined grating. In each trial, observers were asked firstly to align the stimuli from the two eyes; they were then instructed to adjust the position of a reference line to indicate the perceived phase of the binocularly combined grating. Surrounding the gratings, a high-contrast frame (width, 0.11°; length, 6°) with four white diagonal lines (width, 0.11°; length, 2.83°) was always presented during the test to help observers maintain fusion. The gratings had a period of 2 cycles corresponding to 180 pixels, thus the phase adjustment had a step size of 4 degrees of phase per pixel.

### Curve fits

Individuals’ effective contrast ratio at balance point were calculated by fitting the perceived phase (*φ*) versus interocular contrast ratios (*δ*) functions (i.e., the PvR functions), with a modified contrast-gain control model from Huang *et al*.^21^:

1 $$\phi ={\tan }^{-1}[\frac{1-{(\delta /bp)}^{1+\gamma }}{1+{(\delta /bp)}^{1+\gamma }}\cdot \,\tan (\frac{\theta }{2})]$$

In which, *bp* and *γ* are two free parameters. ‘*bp*’ represents the interocular contrast ratio when the two eyes make equally contributions to binocular combination (i.e., the balance point) and ‘*γ*’ represents a non-linear factor. Curve fitting was conducted in Matlab (MathWorks, Natick, MA) using the nonlinear least squares method to minimized ∑(*φ*_*theory*_ − *φ*_*observed*_)^2^.
